# FvKex2 is required for development, virulence, and mycotoxin production in *Fusarium verticillioides*

**DOI:** 10.1007/s00253-024-13022-8

**Published:** 2024-02-22

**Authors:** Limin Wu, Wenyin Bian, Yakubu Saddeeq Abubakar, Jiayi Lin, Huijuan Yan, Huan zhang, Zonghua Wang, Changbiao Wu, WonBo Shim, Guo-dong Lu

**Affiliations:** 1 Fujian Vocational College of Bioengineering, Fuzhou, 350002 China; 2 Key Laboratory of Bio-Pesticide and Chemical Biology, Ministry of Education, Fujian Agriculture and Forestry University, Fujian, Fuzhou, 350002 China; 3 Department of Biochemistry, Ahmadu Bello University, Zaria, 810281 Nigeria; 4 Guangzhou University of Chinese Medicine, Guangzhou, 510006 China; 5 Department of Plant Pathology and Microbiology, Texas A&M University, College Station, TX 77843-2132 USA

**Keywords:** *F. verticillioides*, Fumonisin B1, Kexin-like protein, Pathogenicity, Proprotein convertase

## Abstract

**Abstract:**

*Fusarium verticillioides* is one of the most important fungal pathogens causing maize ear and stalk rots, thereby undermining global food security. Infected seeds are usually unhealthy for consumption due to contamination with fumonisin B1 (FB1) mycotoxin produced by the fungus as a virulence factor. Unveiling the molecular factors that determine fungal development and pathogenesis will help in the control and management of the diseases. Kex2 is a kexin-like Golgi-resident proprotein convertase that is involved in the activation of some important proproteins. Herein, we identified and functionally characterized FvKex2 in relation to *F. verticillioides* development and virulence by bioinformatics and functional genomics approaches. We found that FvKex2 is required for the fungal normal vegetative growth, because the growth of the *∆Fvkex2* mutant was significantly reduced on culture media compared to the wild-type and complemented strains. The mutant also produced very few conidia with morphologically abnormal shapes when compared with those from the wild type. However, the kexin-like protein was dispensable for the male role in sexual reproduction in *F. verticillioides*. In contrast, pathogenicity was nearly abolished on wounded maize stalks and sugarcane leaves in the absence of *FvKEX2* gene, suggesting an essential role of Fvkex2 in the virulence of *F. verticillioides*. Furthermore, high-performance liquid chromatography analysis revealed that the *∆Fvkex2* mutant produced a significantly lower level of FB1 mycotoxin compared to the wild-type and complemented strains, consistent with the loss of virulence observed in the mutant. Taken together, our results indicate that FvKex2 is critical for vegetative growth, FB1 biosynthesis, and virulence, but dispensable for sexual reproduction in *F. verticillioides*. The study presents the kexin-like protein as a potential drug target for the management of the devastating maize ear and stalk rot diseases. Further studies should aim at uncovering the link between FvKex2 activity and FB1 biosynthesis genes.

**Key points:**

*•The kexin-like protein FvKex2 contributes significantly to the vegetative growth of Fusarium verticillioides.*

*•The conserved protein is required for fungal conidiation and conidial morphology, but dispensable for sexual reproduction.*

*•Deletion of FvKEX2 greatly attenuates the virulence and mycotoxin production potential of F. verticillioides.*

**Supplementary Information:**

The online version contains supplementary material available at 10.1007/s00253-024-13022-8.

## Introduction

Maize ear rot, stalk rot, or blight caused by the ascomycete *Fusarium verticillioides* is one of the most serious maize diseases in most maize-growing areas worldwide (Gai et al. 2018). *F. verticillioides* is an aggressive filamentous pathogenic fungus that infects many plants, especially maize, producing a group of carcinogenic mycotoxins called fumonisins (Gelderblom et al. 1988). After infection, the crop becomes maldeveloped and its yield is reduced by 5–10% or, in the worst case, nothing is reaped at harvest time (Desjardins et al. 2002). Pathogens of the *Fusarium* genus use both pathogen-specific and general mechanisms to colonize their hosts. Their virulence factors may include host-specific toxins and proteases, which are also required for fungal development (Zheng et al. 2018).

*F. verticillioides* produces fumonisin B1 (FB1) mycotoxins which contaminate the host grains and are injurious to human health (Deng et al. 2021). Such compounds can lead to human and animal cytopathy, esophagus cancer, and other related diseases, and also damage the liver and kidney functions (Gelderblom et al. 1991; Grenier et al. 2012). FB1 can also cause the softening of horse white matter and pig pulmonary edema (Gelderblom et al. 1988; Grenier et al. 2012; Marasas et al. 1988). Therefore, infested corn may seriously endanger the health of humans and livestock (Desjardins et al. 2002; Gelderblom et al. 1988).

Golgi are organelles found in nearly all eukaryotic cells. Golgi subcellular localization differs among eukaryotes. In *Saccharomyces cerevisiae*, many Golgi apparatuses are distributed in the entire cytoplasm (Nakano and Luini 2010). In mammals, the Golgi apparatus complex is generally near the centrosome and close to the cell nucleus (Kellogg et al. 1995). The Golgi share certain characteristics with endoplasmic reticulum (ER) exit sites among eukaryotes (Suda and Nakano 2012). There are many important metabolic processes that occur in Golgi. In eukaryotic cells, Golgi complete the final processing and packaging of proteins for cell secretions. This occurs through vesicle transport from the ER to the Golgi membrane, the vesicles fuse with the Golgi membrane and release their contents into the Golgi body lumen for further processing (Delic et al. 2013; Abubakar et al. 2023). Some extracellular polysaccharides are also synthesized in the Golgi (Duran et al. 2008).

Modifications of secretory proteins, such as glycosylation, formation of disulfide bonds, and site-specific proteolytic cleavage, are crucial and well conserved in protein maturation in eukaryotic cells (Caballero-Pérez et al. 2021). Precursor proteins are cleaved off the leader pro-peptide by (presumably) late Golgi-resident proteases, such as the Kex2 protease of *S. cerevisiae* (Redding et al. 1991), to be converted to mature and functional forms (Fuller et al. 1989). Kex2 (Kex means killer expression) was first discovered as a proprotein-processing protease in *S. cerevisiae* (Wickner 1974) in which the gene coding for the protein is located on chromosome XIV and the protein contains a Golgi retrieval signal (Wilcox et al. 1992). Kex2 is necessary for the secretion of an active toxin in *S. cerevisiae* (Leibowitz and Wickner 1976). In the presence of a yeast Kex2 protease, an insulin precursor in recombinant production in a yeast (such as *Pichia pastoris*) can be processed into a mature and functional insulin similar as observed by a human processing endopeptidase (Caballero-Pérez et al. 2021; Rhodes et al. 1989).

Kexin-like proteinases, which is a subfamily of subtilisin-like serine proteinases such as mammalian furins and yeast Kex2, mainly mediate site-specific proteolysis in eukaryotic cells in the late secretory pathway (Rockwell et al. 2002; Turpeinen et al. 2013) which activate zymogens of secreted proteinases (Enderlin and Ogrydziak 1994; Newport and Agabian 1997), polysaccharide-degrading enzymes (Goller et al. 1998), and lipases (Pignède et al. 2000). KexB in *Aspergillus fumigatus* is also involved in *N*-glycan processing indirectly due to needs of appropriate protein processing and folding (Wang et al. 2015). A Kex2 mutation in yeast suppresses the functions of vacuolar proton-translocating ATPase (V-ATPase) (Oluwatosin and Kane 1998). KexB in *Aspergilli* is involved in processing the precursor protein of UstA for biosynthesis of ustiloxin B, a known inhibitor of microtubule assembly (Umemura et al. 2014; Yoshimi et al. 2016). Some pathogens lacking the kexin proteins showed impaired pathogenicity (Newport et al. 2003; Richard et al. 2001; Rockwell et al. 2002; Venancio et al. 2002; Wösten et al. 1996). Moreover, disruption of the *KEXB* gene led to abnormal polarized growth in *Aspergillus oryzae*, *Aspergillus nidulans*, and *Aspergillus niger*, morphological abnormalities in *S. cerevisiae*, *Paracoccidioides brasiliensis*, and *Candida albicans* (Komano and Fuller 1995; Newport et al. 2003; Venancio et al. 2002), and increased sensitivity to certain drugs in *Candida glabrata* (Bader et al. 2001). The activities of some cell wall–modifying enzymes were reduced in *KEXB* deletion mutants of *A. fumigatus* (Wang et al. 2015).

We investigated the functions of the Kex2 homolog gene in *F. verticillioides* and analyzed its roles in the pathogenic fungus by pathological, cell biology, and genetic approaches. The hypothetical protein Kex2 (FVEG_03645) from the serine protease family is speculated to code for a putative kexin-like endoprotease. In our study, we found that FvKex2 is critical for vegetative growth, FB1 biosynthesis, and virulence, but dispensable for sexual reproduction in *F. verticillioides.* The findings can be used to further reveal the molecular mechanism of plant *F. verticillioides* interaction*.*

## Materials and methods

### Fungal strains, media, and culture conditions

*F. verticillioides* 7600 (strain A149; mtA^−^; *MAT1-1*; Fungal Genetic Stock Center) was used as the male wild-type strain (Yamamura and Shim 2008) while *F. verticillioides* 7598 (strain A109; mtA^+^; *MAT1-2*; Fungal Genetic Stock Center) was used as the female wild-type strain which function as nucleus donor and nucleus acceptor respectively in this study. For vegetative growth analysis, the wild-type, *Fvkex2* mutant, and CFvKex2 strains were cultured in potato dextrose agar (PDA, Difco, Detroit, MI, USA) medium and incubated at 25 ℃. Radial growth was determined by measuring the fungal colony diameters after 5 days of incubation on PDA at 25 ℃. Conidia production was assayed on V8 medium as previously reported (Shim and Woloshuk 2001). For DNA extraction, hyphae were harvested on YEPD medium (yeast extract 3 g/L, peptone 10 g/L, dextrose 20 g/L, agar 20 g/L); V8 agar and Myro medium (ammonium phosphate NH_4_H_2_PO_4_ 1 g/L, potassium dihydrogen phosphate KH_2_PO_4_ 3 g/L, magnesium sulfate.7H_2_O 2 g/L, sodium chloride 5 g/L, sucrose 40 g/L, adjust pH to 5.9 using phosphoric acid) (Yan et al. 2021). For sexual reproduction assay, we used carrot agar medium (carrot 400 g/L, H_2_O 400 ml agar 20 g/L, autoclaved for 10 min and squeezed through cheese cloth, filled to 1L with ddH_2_O, autoclaved full time for 30 min). *Fusarium* regeneration broth (FRB: sucrose 34.3 g/L, yeast extract 0.02 g/L) and *Fusarium* regeneration agar (FRA: sucrose 34.3 g/L, yeast extract 0.02 g/L agar 1.0 g/L) were used for recovery growth of transformants. LB medium (yeast extract 5 g/L, tryptone 10 g/L, NaCl 10 g/L, pH 7.0) was used for bacterial cultivation.

### Generation of a *FvKEX2* deletion mutant and complementation

The *FvKEX2* gene was knocked out in the mtA^−^ strain 7600 (A149) by a homologous recombination strategy (Supplemental Fig. S1A). To generate a *Fvkex2* mutant, the upstream (A) and downstream (B) fragments of the *FvKEX2* gene were amplified using the primer pairs FvKAF/FvKAR and FvKBF/FvKBR, respectively (primer sequences are listed in Table 1). Fragment A and the vector pCX62 ( Zhao et al. 2004) were both digested with the restriction enzymes *Kpn*I and *Hin*dIII and the digested products were ligated using T4 ligase to obtain a recombination vector Ap. On the other hand, the downstream fragment B and the above recombinant vector Ap were both digested with the restriction enzymes *Bam*HI and *Xba*I, and the products were ligated to obtain another recombinant vector ApB in which the hygromycin B gene sequence (H) was situated between A and B sequences (Supplemental Fig. S1A). The primer pair FvKAF/FvKBR (Table 1) was used to amplify the AHB fragment. The amplicon was separated on agarose gels and the purified AHB fragment was used for transformation into the wild-type protoplasts as reported previously (Sweigard et al. 1995). Transformants were selected on plates containing hygromycin for screening and confirmation. All the primers used in this study are listed in Table 1. The knockout mutants were obtained after screening the transformants by PCR (Supplemental Fig. S1C) and further confirmed by qRT-PCR where the *FvKEX2* transcript could not be detected in the deletion mutants (data not shown). Southern blot analysis was also used to further confirm the deletion of the *FvKEX2* gene (Supplemental Fig. S1B and D). Bacterial plasmids were isolated using a TIANNprep Plasmid Extraction Kit (TIANGEN BIOTECH, Beijing, China). All molecular biology-related techniques, including *Fusarium* transformations, were performed as described previously (Shim et al. 2006; Shim and Woloshuk 2001). For complementation of the *FvKEX2* gene, the complete *FvKEX2* coding sequence (including its native promoter sequence) and the GFP gene sequence were amplified from the genomic DNA of the wild-type A149 and the pMD18-T (TaKaRa, Dalian, China) vector, respectively, by PCR using the primer pairs CFvKF/CFvKR and CGF/CGR (Table 1), respectively, and separated on agarose gels. The purified target bands were digested with *Spe*I, and cloned into the pKNTG vector containing a neomycin resistance gene (Yang et al. 2010). The vector was transformed into Δ*Fvkex2* mutant protoplasts, and the positive transformants were screened from neomycin-containing media and confirmed by PCR using the primer pair CFvKF/CGR (data not shown).

**Table 1:** Primers used in the study and their respective sequences

Primer	Sequence (5′–3′)	Restriction enzyme	Vector
FvKAF	CGGGGTACCGGTCCTGAGAAGGCTGTTGA	*Kpn*I	pCX62 (Zhao et al. 2004)
FvKAR	CCCAAGCTTTTCGCATTTGGTTTGAGACG	*Hin*dIII	pCX62 (Zhao et al. 2004)
FvKTF	GGCTGTCAACGAGGTCATCTAC		
FvKTR	GCCTGCCTTTATTCTTCTGCCTA		
FvKBF	CGCGGATCCCCACCTCCAAATCCGTCTCC	*Bam*HI	pCX62 (Zhao et al. 2004)
FvKBR	GCTCTAGACGCTTCCCATCGCTACAACA	*Xba*I	pCX62 (Zhao et al. 2004)
FvKUA	CCAACTTCACCATCAGCGACTA		
H853-U	GACAGACGTCGCGGTGAGTT		
H598	GGCTCCAACAATGTCCTG		
FvKOF	AAGCGGAGATGCTATTGTAAGT		
FvKOR	CTTGTTGGGTCGTTCAGGGT		
CFvKF	ATGAGGCTGTCAACGAGG		pKNTG (Yang et al. 2010)
CFvKR	GGACTAGTACGCCCGCCCAAAGGTCTT	*Spe*I	pMD18-T (TaKaRa, Dalian, China)
CGF	GGACTAGTATGGTGAGCAAGGGCGA	*Spe*I	pMD18-T (TaKaRa, Dalian, China)
CGR	CGGACTAGTTTACTTGTACAGCTCGTC	*Spe*I	pMD18-T (TaKaRa, Dalian, China)

### Southern blot analysis

Southern hybridization was performed as described in a previous report (Shim and Woloshuk 2001). Briefly, the genomic DNA of *F. verticillioides* was extracted as described by Kim et al. (2005), and was then digested by *Sal*I and separated by agarose gel electrophoresis and transferred onto GelBond® membranes (Sigma-Aldrich, St. Louis, MO, USA). The assay was then conducted using a DIG-High Prime DNA labeling and detection starter kit I (Cat. no. 11745832910, Roche, Mannheim, Germany) following the manufacturer’s protocol. To use as a probe, a sequence-unique segment of the *FvKEX2* gene was amplified from the genomic DNA of A149 using the primer pair FvKTF/FvKTR (Table 1) and labeled with ^32^P (Stratagene, La Jolla, CA, USA).

### Random ascospore analysis

Sexual crosses of *F. verticillioides* targeted strains were performed as described by Klittich and Leslie (1988) and Zhang et al. (2019). Briefly, the conidia of the mtA^+^ strain, *F. verticillioides* A109, spread onto carrot agar plates and incubated for 7 days at 25 °C. Then, conidia (5 × 10^6^ spores) of the mtA^−^ strain A149 and mutant strains generated in this background were applied to plates covered with the A109 strain. After 20 days of incubation at 25 °C, with a 14 h/10 h light/dark cycle and then 10 h of black (near-UV) light at night daily, ascospores were collected and observed from pooled perithecia.

### Conidiation

We inoculated the wild-type and mutant strains on V8 medium and incubated at 28 °C for 5 days. Conidiation was assayed by randomly excising equal portions of the strain colonies using a cork borer (1 cm in diameter), washing these with equal volumes (1 mL) of sterile ddH_2_O by vortexing in sterile tubes, filtering the solution through sterile Miracloths, and counting the spores under a light microscope with the help of a hemocytometer (Shim et al. 2006).

### Virulence assay

Maize line B73 was used for the stalk rot assay as described by Shim et al. (2006). Briefly, stalks from 2-week-old B73 maize (planted at 25 °C in the dark) were wounded with a sterile toothpick and mycelia from the fungal strains were used to infect the wounded areas, respectively. Also, conidia were harvested from the wild-type and *Fvkex2* mutant strains grown on V8 agar plates (at 28 °C for 7 days) and stabilized with 2.5% Tween 60, after which 3 µL of 10^7^ conidia/mL from each strain was dropped onto the wounded sites. The infected maize plants were placed in a dark growth chamber at a temperature of 25 °C for 1 week. Details of the stalk rot and virulence assays were described by Shim et al. (2006) and Kim et al. (2005), respectively. Furthermore, sugarcane leaves were also used to test for virulence of the fungal strains where the wounded areas on the sugarcane leaves were inoculated with the fungal mycelial blocks from solid media. The tested leaves were incubated for 24 h under 95% relative humidity and in the dark at 25 ℃, and then at 25 ℃ in continuous light for 4 days.

### Fumonisin B1 (FB1) assay

The amount of FB1 produced by the fungal strains was determined using a standard procedure described previously (Christensen et al. 2012). Briefly, equal volumes (200 µL) of conidia suspension (5 × 10^4^ spores mL^−1^) from the various fungal strains were used to infect corn kernels (2 g of cracked corn kernels for each treatment), and the infected plants were allowed to thrive for 8 days. FB1 extraction and sample purification methods were described previously (Christensen et al. 2012). High-performance liquid chromatography (HPLC) analyses of FB1 and ergosterol were performed as described (Shim and Woloshuk 1999). FB1 levels were then normalized to ergosterol contents. The experiment was repeated twice with three biological replicates.

### Bioinformatics analyses

The amino acid sequence for the FvKex2 protein obtained from GenBank (accession number: CP114042.1) was aligned with the *S. cerevisiae* Kex2 /YNL238W sequence available in the GenBank. A phylogenetic tree was constructed using Kex2 homolog protein sequences from 10 different species. The tree was constructed using MEGA7 software (Tamura et al. 2011). Multiple sequence analysis was done using the ClustalX software (Tamura et al. 2011). Prediction and analysis of the signal peptides were performed using the online tool SignalIP 3.0 Server (http://www.cbs.dtu.dk/services/SignalP/). Prediction and analysis of transmembrane domains were performed using the online tool TMHMM 2.0 Server (https://services.healthtech.dtu.dk/service.php?DeepTMHMM). The location of proteins in cells was predicted using the online tool ProtComp v6.0 (http://sun1.softberry.com/berry.phtml?Topic=protcompan&group=programs&subgroup=proloc).

## Results

### Identification, domain architecture, and phylogenetic analysis of Kex2 proteins

**Fig. 1:**
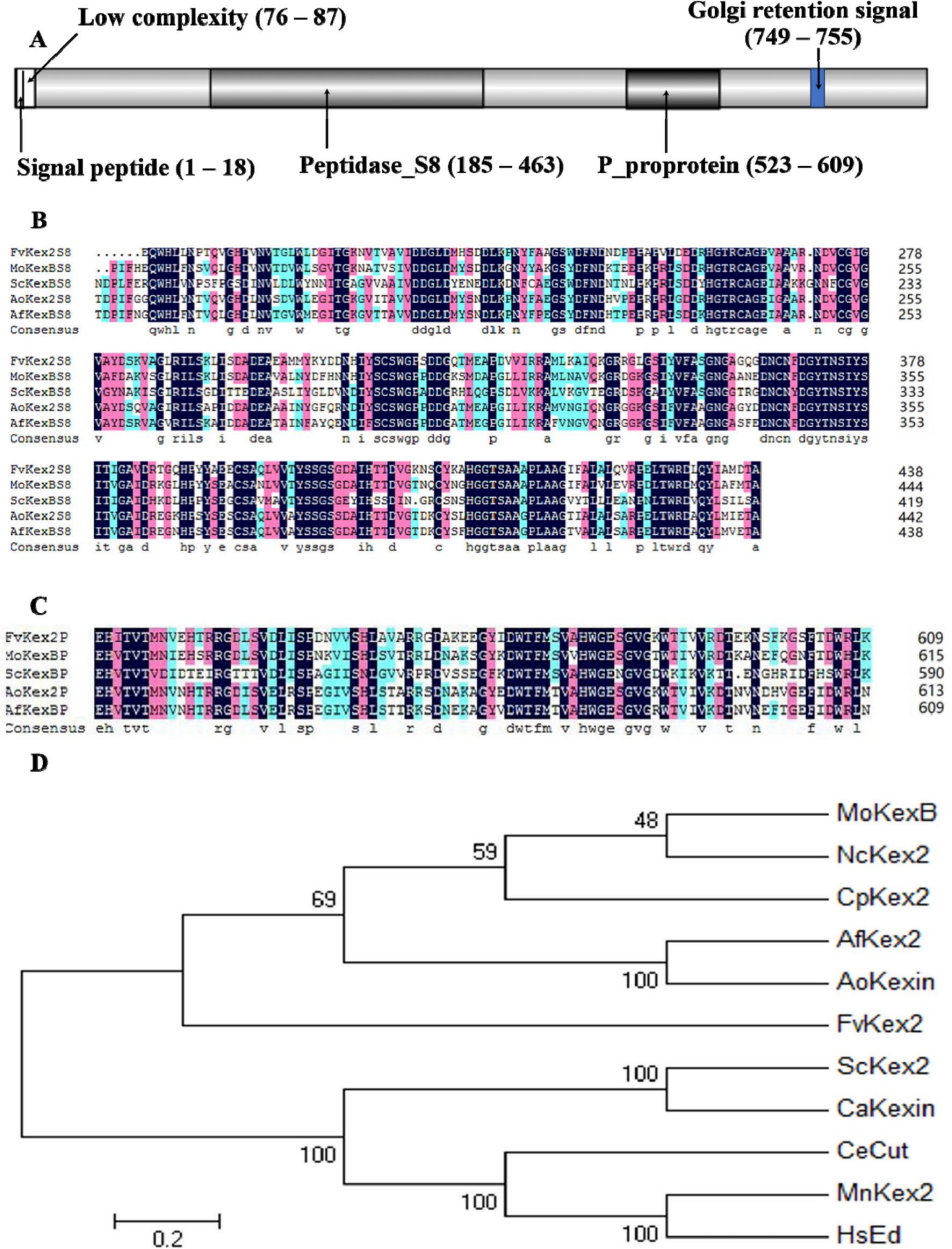
Bioinformatics analyses of Kex2 proteins in phytopathogenic fungi. **A** Domain architechture of FvKex2 protein. The protein has a signal peptide, a Golgi retention signal, and two major domains namely: the peptidase_S8 domain and P_proprotein domain. **B** Sequence alignment of the putative peptidase_S8 domain (185 to 438 aa) found in Kex2 proteins among different fungal species (percentage similarity is 79.23%). **C** Sequence alignment of the putative P_proprotein domain (523 to 609 aa) found in Kex2 protein among different fungal species (percentage similarity is 76.98%). **D** Phylogenetic relationship of Kex2 homologs in *F.* *verticillioides* and some other organisms. FvKex2 has different similarities with its homologs in *Neurospora crassa* (74%), *Magnaporthe oryzae* (73%), *Cryphonectria parasitica* (73%), *Aspergillus fumigatus* (68%), *Aspergillus oryzae* (66%), *Saccharomyces* *cerevisiae* (62%), *Mus musculus* (57%), *Homo sapiens* (57%), *Candida* *albicans* (55%), and *Caenorhabditis elegans* (52%). Accessions: FvKe2p, CP114042.1; MoKexBp, XP_003716137; ScKexBp, YNL238W; AoKex2p, OOO04277.1; AfKexBp, XP_751534.1; NcKex2, XP_011393111.1; CpKex2, ABB30244.1; CaKexin, KHC60202.1; CeCut, AAA98752.1; MmKex2, EDL25664.1; HsEd, XP_047283818.1.

To identify the *F. verticillioides KEX2* gene, we searched through the fungal genome database (Fungi DB database) at https://fungidb.org using the *S. cerevisiae KEX2* sequence as a query. The search result identified the open reading frame of FVEG_03645 which was annotated to encode a hypothetical protein of 847 amino acid residues. At the putative cytosolic tail of this protein, we identified the peptide sequence 749-YEFELI-755, which conforms to the consensus sequence of the *S. cerevisiae* Kex2 Golgi retention signal YXFXXI (Redding et al. 1991; Wilcox et al. 1992), where X is any given amino acid. In *F. verticillioides*, the result was a protein with 847 amino acid residues, possessing a signal peptide (1–18 aa), a peptidase_S8 domain (185–463 aa), a P_proprotein domain (253–609 aa), and a Golgi retention signal (749–755 aa) (Fig. 1A). We therefore named the identified protein in *F. verticillioides* as FvKex2.

Analysis of FvKex2 homologs in different species by multiple sequence alignment revealed that the Kex2 proteins have in common a conserved peptidase_S8 domain and a P_proprotein domain (Fig. 1A, B, and C). The protein sequences from five fungal species (*F.* *verticillioides* Kex2, *Magnaporthe oryzae*, *S.* *cerevisiae*, *A.* *oryzae*, and *A.* *fumigatus*) share sequence similarities of up to 79.23% and 76.98% for the peptidase_S8 and P_proprotein domains, respectively. Phylogenetic analysis revealed that the FvKex2 protein in *F.* *verticillioides* has close similarity with that in *Neurospora crassa* (74%), revealing a close genetic relationship; its similarity with *Caenorhabditis elegans* Kex2 protein is 52%, which reveals a more distant genetic relationship (Fig. 1D).

### FvKex2 is important for vegetative growth of F. verticillioides

**Fig. 2:**
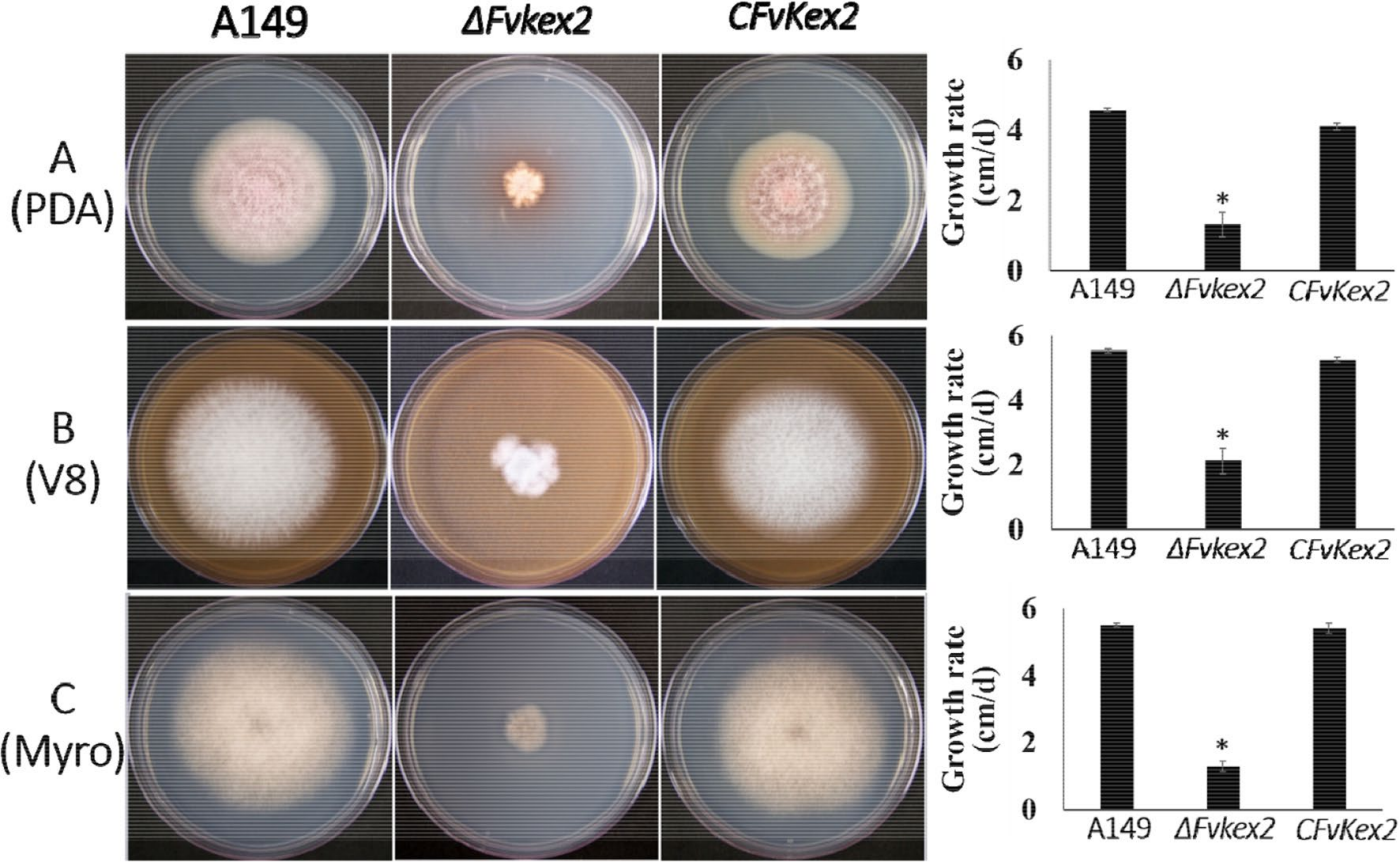
Vegetative growth of *Fvkex2* mutant of different media. The A149—wild type, *∆FvKex2*, and complementation strains were grown in PDA, V8, and Myro media at 25 ℃ for 7 days. Values are presented as mean ± standard deviation from three biological replicates. Asterisks denote significant differences at *p* < 0.05.

To establish the role of FvKex2 in the vegetative growth of *F. verticillioides*, the wild-type A149, the *∆Fvkex2* mutant, and the complemented CFvKex2 strain were grown on PDA, V8, and Myro media, respectively. After 5 days of incubation at 25 ℃, we observed a significant decrease in the mycelial growth of the *ΔFvkex2* mutant on the three different media compared to the wild-type and complementation strains (Fig. 2A, B, and C). This simply suggests that FvKex2 is involved in the normal vegetative growth of *F. verticillioides*.

### The *F. verticillioides* Kex2 protein is required for normal conidiation and conidial morphology

**Fig. 3:**
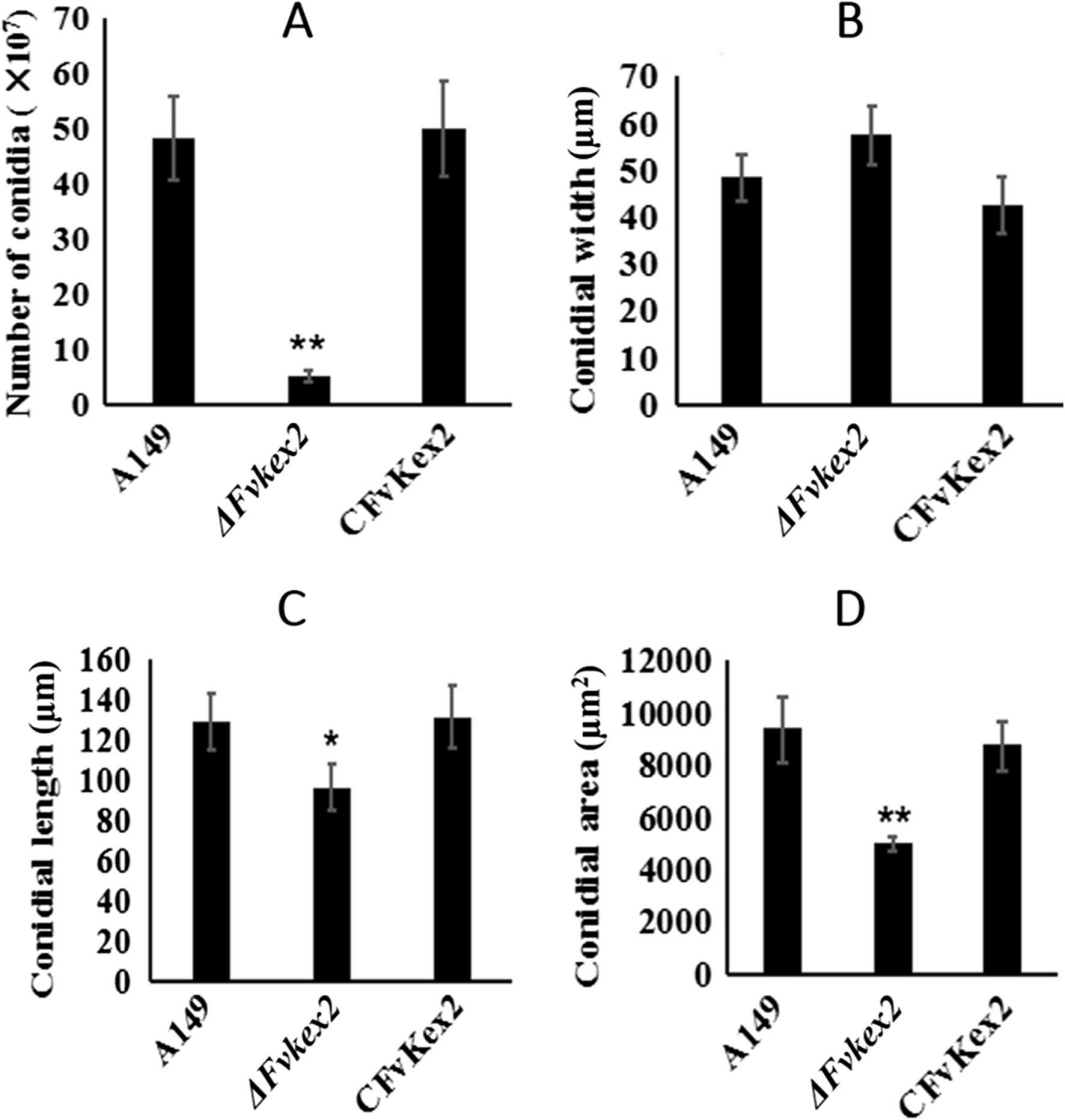
Effects of *FvKEX2* deletion on conidiation and conidial morphology. **A** The number of conidia produced by the *ΔFvkex2* mutant was significantly less than those produced by the wild-type and complemented strain. **B** The mutant conidia had larger widths than those of the wild-type and complemented strains, but the differences were not statistically significant. **C** The conidia harvested from the *ΔFvkex2* mutant were shorter in length than those harvested from the wild-type and complemented strains cultured under the same experimental conditions. **D** Deletion of *FvKEX2* gene caused significant reduction in the surface area of the mutant conidia compared to the wild-type and complemented strains. All experiments were repeated three times and each sample was run in triplicate in each experiment. Values represent mean ± standard deviation. Asterisks show significant differences at* p* < 0.05 for * and *p* < 0.01 for **.

To further understand the role of FvKex2 on the asexual development of the fungus, we harvested and compared the number of conidia produced by the three strains under the same laboratory conditions. Similar to vegetative growth, we found a significant decrease in the number of conidia produced by the *∆Fvkex2* mutant compared to the control strains (Fig. 3A). The number of conidia produced by the *∆Fvkex2* mutant was only about 10% of those produced by the wild-type strain. We also measured the widths, lengths, and average areas of the conidia from the various strains. The results revealed that the *∆Fvkex2* mutant conidia were about 18.61% wider than the wild-type conidia, although the difference was not statistically significant (Fig. 3B). Lengthwise, the mutant conidia were however significantly shorter than those from the wild-type and complemented strains (Fig. 3C, Supplemental Fig. S2). In addition, the average area of a *Fvkex2* mutant conidium was found to be about 46.87% smaller than the wild-type conidia (Fig. 3D). Taken together, these results suggest that FvKex2 contributes to normal conidiation and conidial morphology of *F. verticillioides*.

### FvKex2 is dispensable for sexual reproduction

**Fig. 4:**
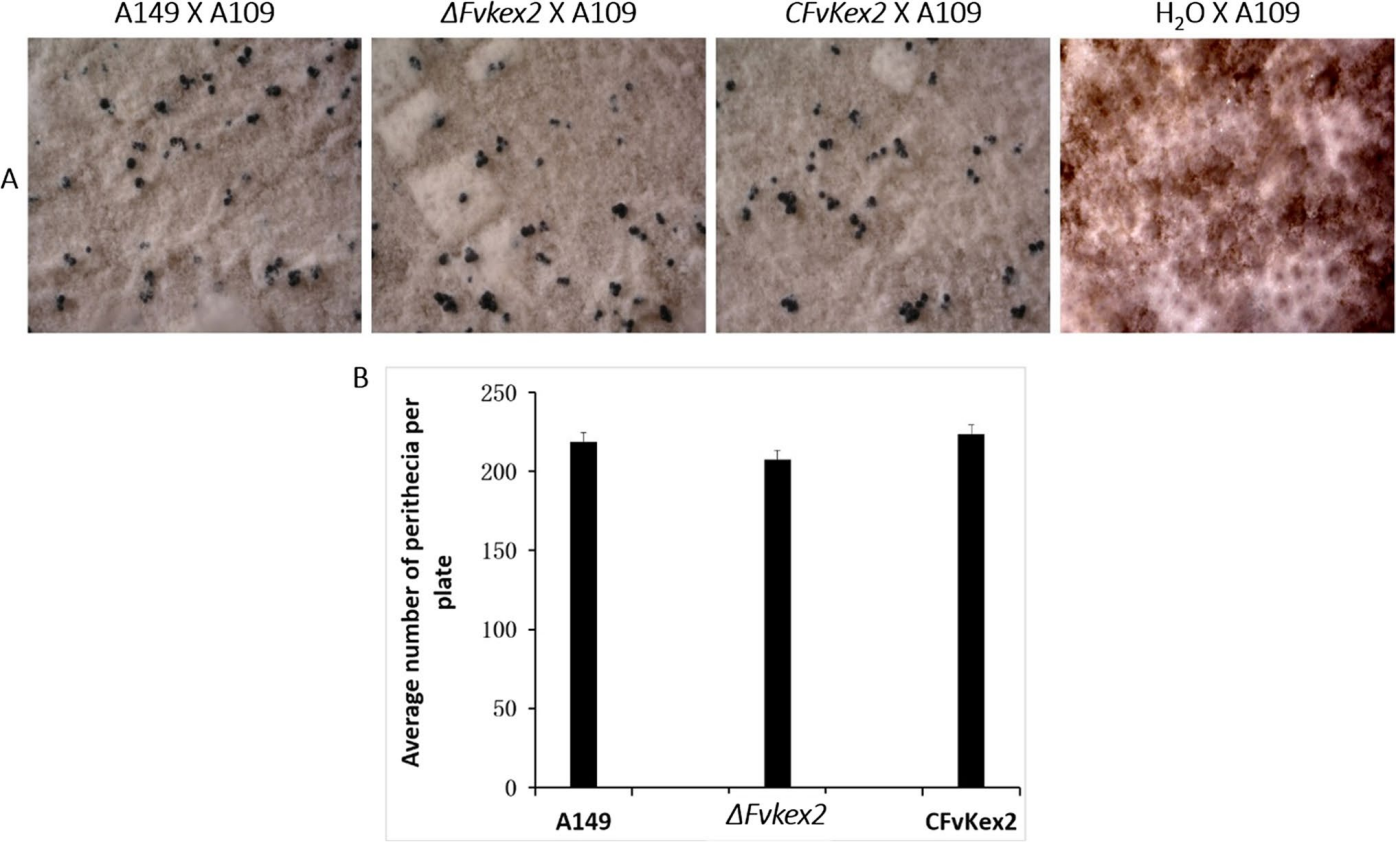
Role of *FvKEX2* in sexual development of *F. verticillioides. ***A** The wild-type (A149), the *∆Fvkex2* mutant, and CFvKex2 were crossed as males with a sexually compatible wild-type strain (A109) acting as female. The amount of perithecia induced by the mutant was similar to those produced by the controls. No perithecia were formed in a cross between the negative control (H_2_O) and A109. **B** There were no significant differences (*p* < 0.05) in the average number of perithecia per plate induced by the mating with the *∆Fvkex2* mutant when compared to the wild-type and complemented strains. Values represent mean ± standard deviation. Each strain was tested in triplicate. Perithecia of the different crosses were controlled for content of ascospores but no differences in spore numbers were observed (data not further shown).

*F. verticillioides* is well-known to reproduce both sexually and asexually (Zhang et al. 2019). Since we found FvKex2 to be important for the fungal asexual development (Figs. 2 and 3), we intended to further investigate its possible role in the sexual development of *F. verticillioides*. To achieve this, we carried out sexual crosses of the various strains and subsequently observed and compared the number of perithecia produced by the various fungal strains. The results revealed no obvious difference in the number of perithecia induced by the mtA^−^ wild type, CFvKex2, and *∆Fvkex2* strains after crossing as males with the sexually compatible mtA^+^ strain A109 (Fig. 4A and B). Because we observed that the number of ascospores in the perithecia of the various strains was similar (data not shown), we did not proceed with the ascospore quantification. These results suggest that FvKex2 does not contribute to the male function in the sexual development of *F. verticillioides*.

### FvKex2 is essential for pathogenicity of *F. verticillioides*

**Fig. 5:**
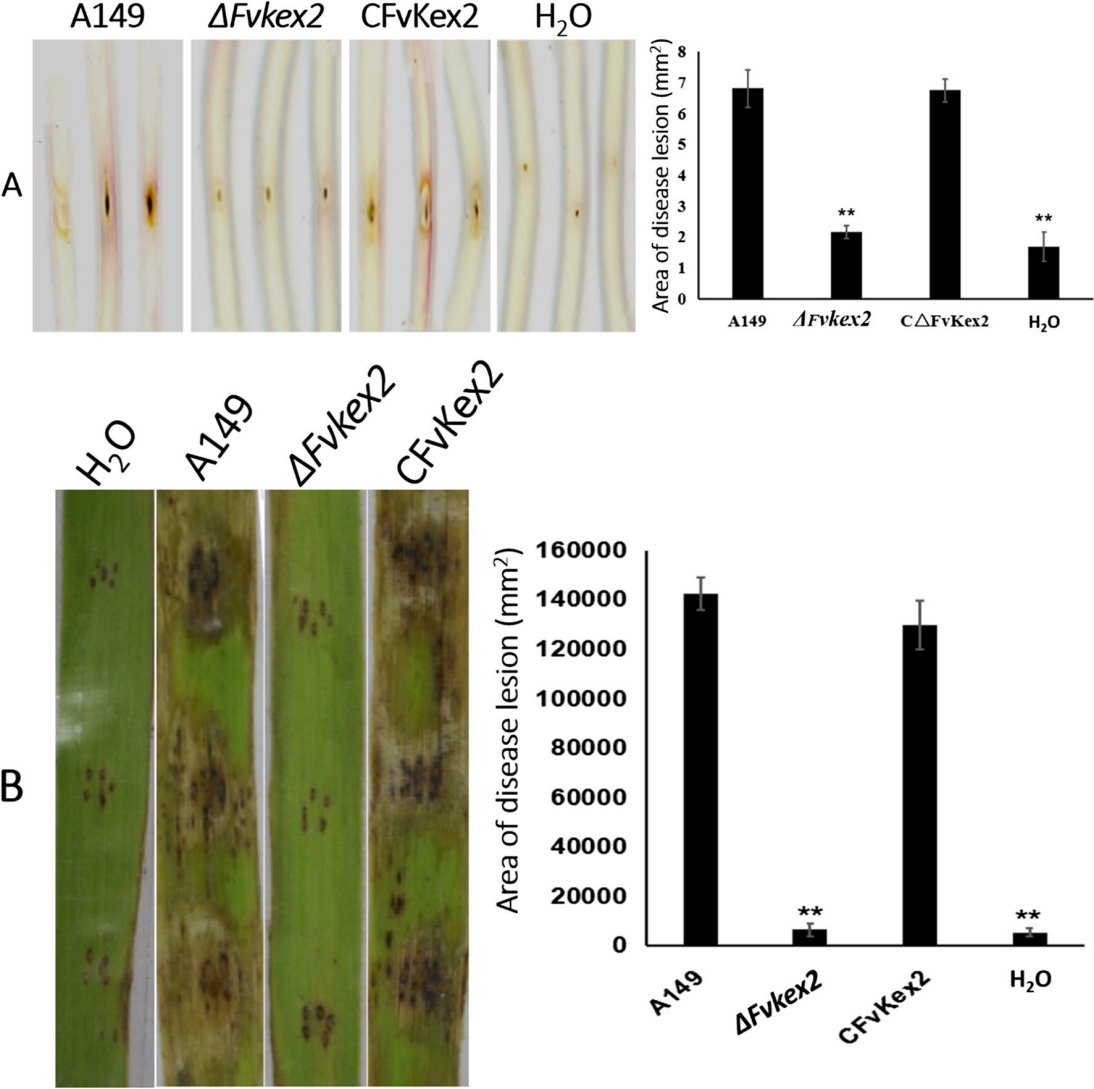
Effect of *FvKEX2* deletion on the fungal virulence. **A** The *ΔFvkex2* mutant was able to cause very weak stalk rot symptoms on maize stalks compared to the positive controls. **B** The pathogenicity of the *∆Fvkex2* mutant was also abolished on sugarcane leaves, different compared to the wild-type and complemented strains. Values are presented as mean ± SD from three independent experiments. In each experiment, three apparently healthy leaves were used for each treatment. Asterisks denote significant differences at *p* < 0.01.

A previous study has demonstrated that Kex2 is important for the pathogenicity of *C. albicans* in the mouse model (Newport et al. 2003). We therefore predicted that the functions of this protein could be conserved in eukaryotes and hence hypothesized that FvKex2 is critical for *F. verticillioides* virulence. To test this hypothesis, about 3 μL of conidia suspension (10^7^ conidia/mL) from each strain was used to infect healthy stalks of maize, respectively, using a microinjector. At 5 days post-infection, we observed that the *∆Fvkex2* mutant could not develop any significant stalk rot lesion on the stalk tissues in comparison with the wild-type and the complemented strain (Fig. 5A), suggesting a loss of virulence due to *FvKEX2* gene deletion. To further verify this, we used sterile needles to create small wounds on sugarcane leaves and infected the leaves with mycelial blocks of similar dimensions from the wild-type, the *∆Fvkex2* mutant, and the complemented strain. Consistent with the virulence on maize stalks, we found that the *∆Fvkex2* mutant failed to develop any observable sugarcane leaf blight symptoms as seen on the leaves infected with the wild-type and complemented strains (Fig. 5B). Put together, we infer here that FvKex2 is required for the virulence of *F. verticillioides*.

### FvKex2 is required for FB1 biosynthesis in F. verticillioides

**Fig. 6:**
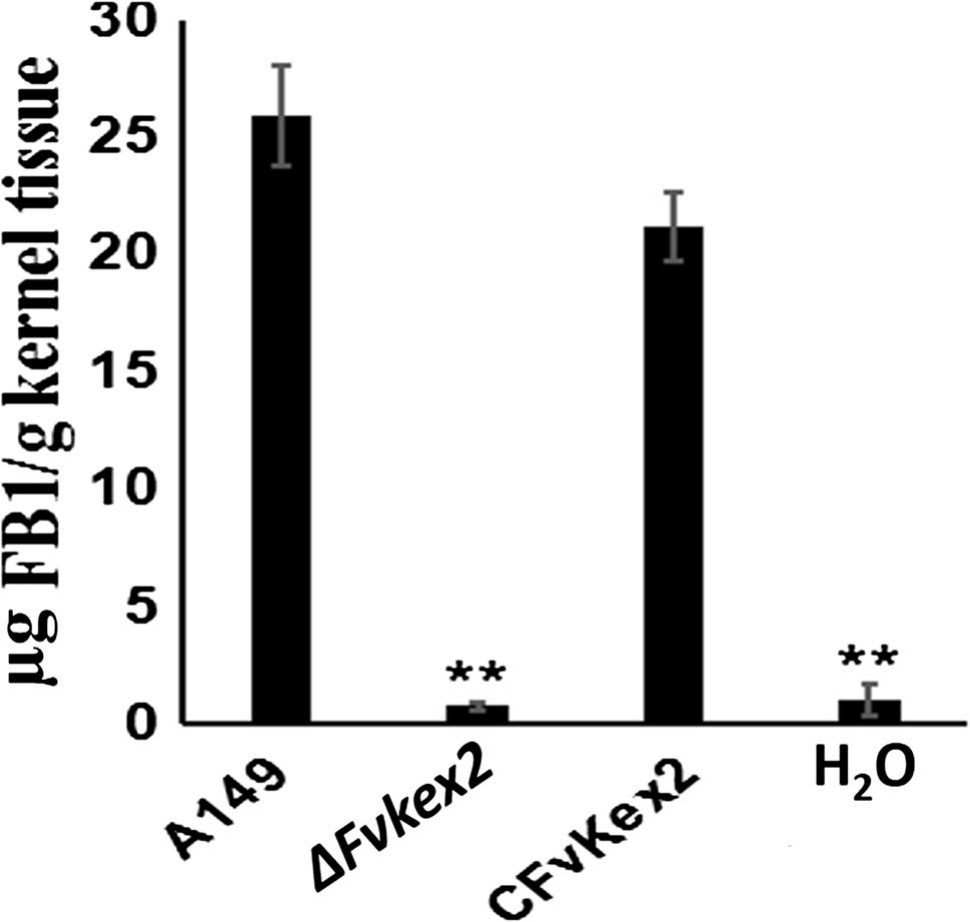
Role of FvKex2 in fumonisin B1 (FB1) production in *F. verticillioides. *The amounts of FB1 produced by each strain in infected maize kernels were quantified 8 days after inoculation. FB1 and ergosterol were quantified by HPLC. The ergosterol level in each sample was used to normalize FB1 levels, thus resulting in relative FB1 production in maize kernels. The mycotoxin produced by the *ΔFvkex2* mutant was significantly less than those produced by the wild-type and complemented strain. Values are presented as mean ± SD from three independent infection experiments; in each experiment, 2 g of cracked corn kernels was used for each treatment. Asterisks denote significant differences at* p* < 0.01.

FB1 mycotoxin is what makes *F. verticillioides*–infested food and feeds a threat to human and animal health (Gelderblom et al. 1988; Nelson et al. 1993). Therefore, we decided to investigate whether FvKex2 has any contributory role to FB1 production in the fungus. To test this, we inoculated cracked corn kernels with equal volumes of conidia suspension (5 × 10^4^ spores mL^–1^) from the wild-type, *∆Fvkex2* mutant, and CFvKex2 strains and incubated the infected plants for 8 days, after which the infected tissues were collected and subjected to FB1 analysis by HPLC. We observed that the amounts of FB1 produced by the wild-type and the complemented strain were 32-fold higher than that produced by the *∆Fvkex2* mutant (Fig. 6). From these results, we conclude that FvKex2 is essential for normal FB1 production in *F. verticillioides*.

## Discussion

Kex2 is a subtilisin-like serine protease that requires Ca^2+^ for its functions and is resident in the Golgi. It is implicated in post-translational modification (proprotein processing) which is an important biological process of protein maturation (Gómez-Gaviria et al. 2020). It carries out its function at the trans-Golgi network (TGN) and it cycles between Golgi vesicles and late endosomes (Bryant and Stevens 1997). Unlike insects and animals, *F. verticillioides* possesses only one kexin protein similar to what was observed in *S. cerevisiae*, *A. nidulans*, and *A. oryzae* (Rockwell et al. 2002). The *FvKEX2* gene is located on chromosome II and its product is a hypothetical protein localized to the Golgi membrane. Our bioinformatics analysis revealed that the FvKex2 protein contains a signal peptide, and the protein is a member of the serine protease family.

Multiple sequence alignment and an evolutionary tree showed that *F. verticillioides* Kex2 is most closely related to the *N. crassa* Kex2 protein; hence, they could have similar biological functions. Generally, the *KEX2* gene appears conserved in eukaryotes. Particularly, the S8- and P-domain sequences are also conserved among *F. verticillioides* and the other four fungi considered in this study (*M. oryzae*, *A. oryzae*, *A. fumigatus*, *S. cerevisiae)* and the similarities reach about 79.23% and 76.98%, respectively.

Consistent with our findings, a previous study demonstrated that the Kex2 protein in *C. albicans* can complement the functions of Kex2 in *S. cerevisiae* or *Schizaccharomyces pombe* (Newport and Agabian 1997). Similarly, the Kex2 protein in *A. oryzae* was shown to complement the functions of its ortholog in *A. nidulans* and vice versa (Mizutani et al. 2009). It can therefore be inferred from our bioinformatics analyses and the results from previous findings that the functions of Kex2 proteins are very conserved in fungi.

From our phenotype analyses, we found that the *Fvkex2* mutant showed a significantly reduced vegetative growth with abnormal hyphal polarity compared to the wild-type. The asexual development of the fungus was also affected due to the deletion of *FvKEX2*, as the mutant only produced morphologically abnormal conidia that were about 90% less than those produced by the wild-type and complemented strain. This is similar to the phenotype of *kexB* mutants in *A. niger*, *A. oryzae*, *A. nidulans*, and *A. fumigatus* (Mizutani et al. 2004, 2009; Punt et al. 2003; Wang et al. 2015).

Fungal cell walls are composed of β-glucans, mannan in cell wall mannoproteins and chitin, etc., which help the fungi to resist osmotic pressure and mechanical force to ensure the protection of internal structures (Klis et al. 2002). The main component of the fungal cell wall is chitin, which is synthesized by the enzyme chitin synthase (CS). CS is usually in the form of a zymogen in the cell and is transformed into a viable enzyme by means of proteolytic enzyme activation (Takeshita et al. 2006). In filamentous fungi, CS genes are of 7 classes: class I to VII. Single deletion of classes I, , IV, and VII CS genes usually does not result in any significant phenotypic defects, but deletion of classes III, V, or VI genes often leads to obvious defects, indicating that classes III, V, and VI CS genes play important roles in filamentous fungi (Munro et al. 2003; Soulié et al. 2006; Takeshita et al. 2006). The phenotypic defects of *kex2* mutants are said to be largely due to blockage of cell wall synthesis as a result of Kex2 inactivation negatively influencing the activities of certain cell wall–modifying enzymes (Wang et al. 2015). Therefore, we speculate that FvKex2 may participate in the proper synthesis of classes III, V, and VI CS in filamentous fungi. To clearly address this speculation, further studies need to identify the specific pathway of FvKex2 protein activity and unveil its primary substrates.

*S. cerevisiae* Kex2 is necessary for the secretion of a bioactive toxin (Leibowitz and Wickner 1976). Similarly, we found herein that the *∆Fvkex2* mutant also showed a defect in FB1 production as the production of this mycotoxin was very low compared to the amount produced by the wild-type under the same experimental conditions. There are two possible explanations for this. First, the mutant mycelial growth was slower than that of the wild-type, leading to a lower yield of FB1 mycotoxin. Secondly, the FvKex2 protein may play a major role in FB1 secretion or may be directly involved in the regulation of FB1 expression in *F. verticillioides*.

Previous studies indicated that *kex2* mutants in *C. albicans* have impaired virulence (Newport et al. 2003; Rockwell et al. 2002; Venancio et al. 2002). Here, the *KEX2* orthologue mutant in *F. verticillioides* was similarly observed to lose its pathogenicity on both maize stalk and sugarcane leaves. Since the growth rate of the mutant was adversely affected, the reduced virulence may be attributed to reduced colonization ability in host tissue, coupled with the effects of the host immune responses (Muimba-Kankolongo and Bergstrom 2011).

In ascomycetous yeasts, Kex2 participates in the maturation of the α-mating factor precursor (Fuller et al. 1989; Julius et al. 1984). *S. cerevisiae* and *C. albicans* have two mating types, MATa and MATα (Hull and Johnson et al. 1999) and MTLa and MTLα (Magee et al. 2002), respectively. Kex2 is involved in processing pheromone Mfα in the *S. cerevisiae* mating type MATα strain (Fuller et al. 1989; Panwar et al. 2003). Therefore, in *S. cerevisiae* as also in *C. albicans*, *kex2* mutants of the MTLα and the MTLα mating type, respectively, lost their mating ability but those of the MATa and MTLa mating type expressing the alternate Kex2-independent MATa pheromone Mfa could mate efficiently (Chan et al. 1983; Magee et al. 2002). Homologous genes to the yeast α-factor pheromone gene were found in filamentous ascomycetes, such as *N. crassa*, *Cryphonectria parasitica*, and* M. grisea*, which were predicted to encode the precursor polypeptides that are processed by Kex2 (Bobrowicz et al. 2002; Zhang et al.^1998^; Shen et al. 1999). The expression of these pheromone precursor genes is mating-type-specific. For example, the *mat A-1* gene for a mating-type-specific MATα-HMG-box domain transcription factor controlling the expression of the α-factor-like pheromone is exclusively present and expressed in *mat A* strains but not in *mat a* strains of *N. crassa* (Bobrowicz et al. 2002). In line, deletion of the α-sex pheromone *Mf1-1* precursor gene or silencing of *Kex2* significantly diminishes the male fertility of *C. parasitica*. However, the *Mf1-1* deletion strain was female-competent in contrast to fully silenced *Kex2* strains that as females in crosses with functional male strains gave rise to only barren perithecia (Turina et al. 2003; Jacob-Wilk et al. 2009). In *Fusarium graminearum*, it has previously been reported that deletion of the α-factor pheromone precursor gene *ppg1* reduced male fertility in outcrossings (Lee et al. 2008). In *F. verticillioides*, the 7600 strain (*MAT1-1*) contains a *MAT1-1–1* gene encoding a mating-type-specific MATα-HMG-box domain transcription factor (ID 7249; https://mycocosm.jgi.doe.gov/Fusve1/Fusve1.home.html) like in MAT1-1 strains of other *Fusarium* species and the mating type gene *mat A-1* in the *mat A* strains of *N. crassa* (Ferreira et al. 1998; Yun et al. 2000). Protein Mat1-1–1 in *F. verticillioides* will be required for expression of its Kex2-dependent α-factor-like pheromone precursor gene (Martin et al. 2011) as protein mat A-1 is required for expression of the α-factor-like pheromone precursor gene *ccg-4* (*ppg1*) in *N. crassa* (Bobrowicz et al. 2002). Interestingly, conidia of a *Δccg-4 mat A* strain were unable to act as males and did not fuse with female-specific hyphae (trichogynes) of the opposite mating type *mat a* (Kim and Borkowich 2006). However, in our study, the *∆Fvkex2* mutant showed normal sexual reproduction when acting as a male. Whether *ΔFvkex2* mutants when acting as females in contrast are sterile is still unknown. The involvement of FvKex2 in the female role of the mating mechanism of *F. verticillioides* needs to be investigated in future studies.

## Supplementary Information

Below is the link to the electronic supplementary material.

Supplementary file1 (PDF 152 KB)

## Data Availability

All data generated or analyzed during this study are included in this article.
