# Supplementary material for: FvKex2 is required for development, virulence, and mycotoxin production in Fusarium verticillioides
Source: Appl Microbiol Biotechnol. 2024 Feb 22;108:228. doi: 10.1007/s00253-024-13022-8 (PMC10884074; doi:10.1007/s00253-024-13022-8)
Supplement: Supplementary file 1 [file 253_2024_13022_MOESM1_ESM.pdf]

FvKex2 is required for development, virulence and mycotoxin production in *Fusarium verticillioides*

Limin Wu[1], Wenyin Bian[2], Yakubu Saddeeq Abubakar[2], Jiayi Lin[3], Huijuan Yan[4], Fang zhang[4], Zonghua Wang[2], Changbiao Wu[1], WonBo Shim[4*], Guo-dong Lu[2*]

[1]Fujian Vocational College of Bioengineering, Fuzhou 350002, China

[2]Key Laboratory of Bio-pesticide and Chemical Biology, Fujian Agriculture and Forestry University, Fujian, Fuzhou 350002, China

[3]Guangzhou University of Chinese Medicine, Guangzhou 510006, China

[4]Department of Plant Pathology and Microbiology, Texas A&M University, College Station 77843-2132, TX, USA

*Corresponding author:

Won Bo Shim E-mail address: wbshim@tamu.edu,

Guo-dong Lu

Tel: +86-13950480067 Fax: +86-591-83768251

E-mail address: lgd@fafu.edu.cn

Supplemental Figures

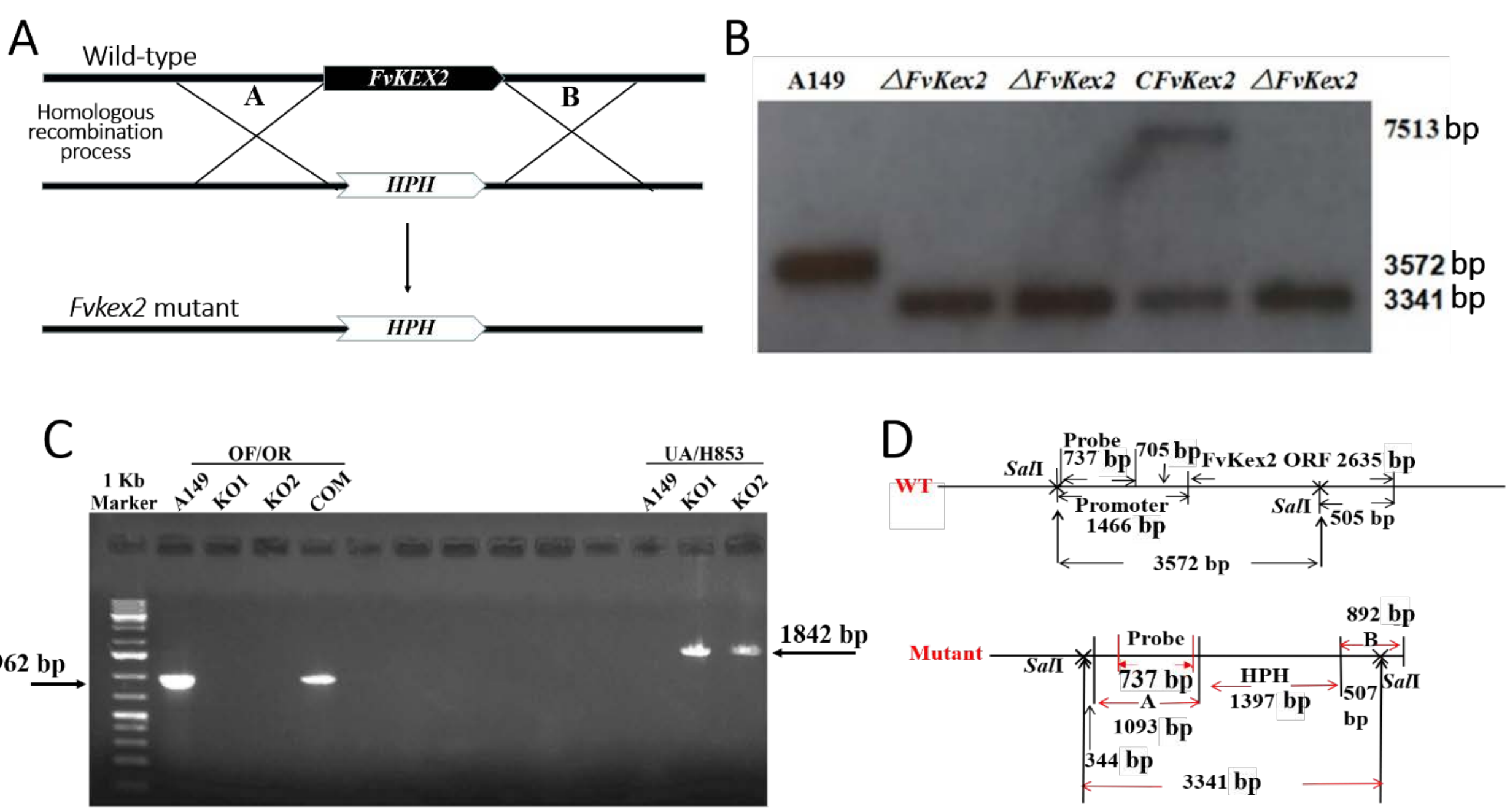

Fig. S1: Targeted *FvKEX2* gene deletion in *F. verticillioides*

(A) Targeted gene deletion strategy for the *FvKEX2* gene. The *FvKEX2* gene (962 bp) was replaced by the hygromycin phosphotransferase (*HPH*) gene in the mutant. (B) Southern blot analysis was used to confirm the *FvKEX2* gene deletion and complementation. Genomic DNA from the indicated strains was digested with *Sal*I and separated on an 1% agarose gel. The target bands of 3572, 3341 and 7513 bp were observed in the wild-type A149, mutant and complemented strain, respectively. **(**C) Confirmation of *FvKEX2* gene deletion by PCR. The FvKOF and FvKOR primers were used to amplify a 962 bp fragment of the gene ORF (open reading frame) using genomic DNA from the indicated strains; the band could not be amplified from the mutant DNA. Also, the primers FvKUA (which anneals upstream of the A fragment) and H853 (which anneals within the *HPH* fragment) gave positive bands of 1842 bp in the mutants, which the band was absent in the wild-type. (D) A map showing the digestion fragments for Southern blot assay. *Sal*I digestion produces 3573 bp and 3341 bp fragments in the wild-type and mutants, respectively.

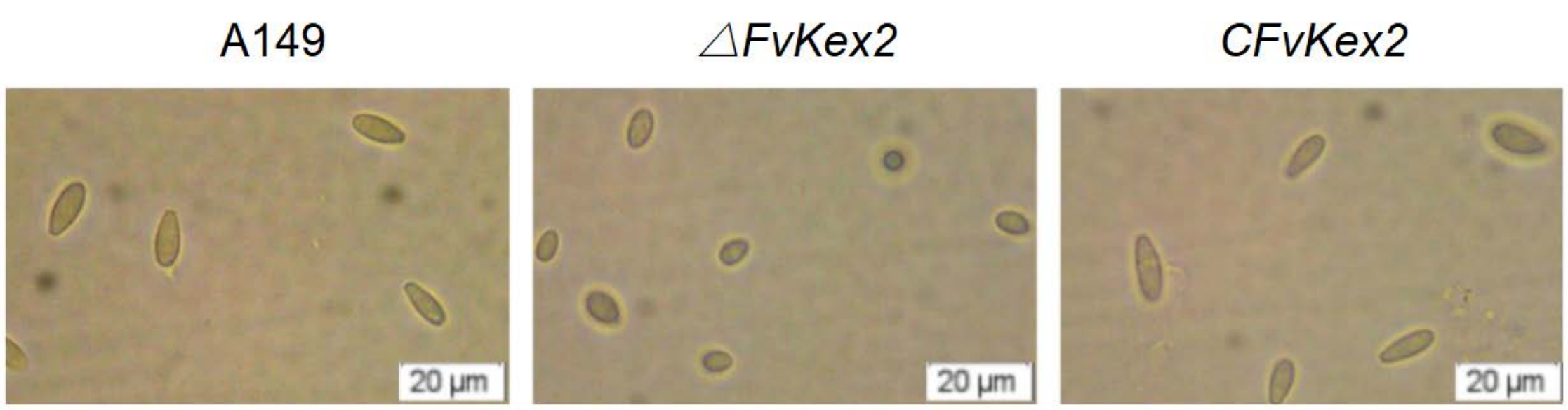

Fig. S2: **Morphology of *Fvkex2* mutant conidia.**

The *ΔFvkex2* mutant has shorter conidia than A149 and *CFvKex2*
